# Ultrasound Phenotype-Based Approach to Treatment Choice in Osteoarthritis

**DOI:** 10.3390/life15071140

**Published:** 2025-07-19

**Authors:** Rositsa Karalilova, Velichka Popova, Konstantin Batalov, Dimitar Kolev, Lyatif Kodzhaahmed, Dimitrina Petrova-Stoyankova, Nikola Tepeliev, Tsvetelina Kostova, Lili Mekenyan, Zguro Batalov

**Affiliations:** 1 Department of Propedeutics of Internal Diseases “Prof. Dr. Anton Mitov”, Medical University of Plovdiv, 4001 Plovdiv, Bulgaria; rositsa.karalilova@mu-plovdiv.bg (R.K.); velichka.popova@mu-plovdiv.bg (V.P.); konstantin.batalov@mu-plovdiv.bg (K.B.); dimitar.kolev@mu-plovdiv.bg (D.K.); lyatif.kodzhaahmed@mu-plovdiv.bg (L.K.); dimitrina.petrova@mu-plovdiv.bg (D.P.-S.); nikola.tepeliev@mu-plovdiv.bg (N.T.); tsvetelina.kostova@mu-plovdiv.bg (T.K.); zguro.batalov@mu-plovdiv.bg (Z.B.); 2 Clinic of Rheumatology, University Hospital “Kaspela”—Plovdiv, 4001 Plovdiv, Bulgaria

**Keywords:** osteoarthritis, clinical phenotypes, ultrasound

## Abstract

**Introduction/Objectives:** Osteoarthritis (OA) is a chronic systemic disease that affects the entire array of joint structures. It is one of the most common chronic, socially significant diseases, associated with a decline in the quality of life of patients and constantly increasing the cost of treatment. Clinical trial outcomes are largely inconclusive, and OA remains one of the few musculoskeletal diseases without an established disease-modifying therapy. One potential explanation is the use of ineffective tools for OA classification, patient stratification, and the assessment of disease progression. There is growing interest in musculoskeletal ultrasonography (MSK US), as it enables the dynamic visualization of the examined structures and gives information about both inflammatory and structural changes that have occurred. Determining the leading ultrasound phenotype, which depends on the most damaged tissue at a given time (bone, cartilage, synovial membrane, joint capsule, ligaments, tendons, menisci, etc.), can rationalize therapy use by selecting patients more suitable for specific treatments. This article aims to evaluate and summarize the potential of MSK US in the process of determining the clinical phenotype of OA and to emphasize the importance of this imaging modality in evaluating further therapeutic strategies. **Method:** A single-center prospective study conducted in the period of September 2023–June 2024 enrolled 259 consecutive patients with proven OA. The statistical program Minitab version 22.2.1 (2025) was used to analyze the data. The predominant and secondary phenotypes were tabulated for each OA localization and were presented numerically and as relative proportions (%). The rate of the most frequently occurring phenotypes was compared against that of the less frequent ones through paired z-tests. The initially acceptable type I error was set at 5%; it was further adjusted for the number of comparisons (Bonferroni). **Results:** The most frequent and predominant US phenotype for patients with knee OA was intra-articular effusion (*n* = 47, 37.90%). It was significantly higher compared to the rest of the US phenotypes: synovial proliferation (*n* = 22, 17.70%; *p* < 0.001), cartilage destruction (*n* = 26, 21%; *p* = 0.001), altered subchondral bone (*n* = 8, 6.50%; *p* < 0.001), extra-articular soft tissue changes (*n* = 12, 9.70%; *p* < 0.001), crystal deposits (*n* = 6, 4.8%; *p* < 0.001), and post-traumatic (*n* = 3, 2.40%; *p* < 0.001). The most common US phenotype for hip OA was altered subchondral bone (*n* = 32, 47.1%), with significant differences from intra-articular effusion (*n* = 12, 17.60%; *p* = 0.001), synovial proliferation (*n* = 5, 7.40; *p* = 0.001), cartilage destruction (*n* = 12, 17.60%; *p* = 0.001), extra-articular soft tissue changes (*n* = 3, 4.40%; *p* = 0.001), crystal deposits (*n* = 3, 4.40%; *p* = 0.001), and post-traumatic (*n* = 0). Altered subchondral bone was also the leading US phenotype for hand OA (n = 31, 55.40%), with significant differences compared to intra-articular effusion (*n* = 1, 1.80%; *p* < 0.001), synovial proliferation (*n* = 7, 12.50%; *p* < 0.001), cartilage destruction (*n* = 11, 19.60%; *p* < 0.001), extra-articular soft tissue changes (*n* = 2, 3.60%; *p* < 0.001), crystal deposits (*n* = 3, 5.40%; *p* < 0.001), and post-traumatic (*n* = 1, 1.80%, *p* < 0.001). For shoulder OA, extra-articular soft tissue changes were the most frequent (*n* = 8, 46.20%), followed by post-traumatic (*n* = 4, 30.70%), as the rate of both phenotypes was significantly higher compared to that of intra-articular effusion (*n* = 0), synovial proliferation (*n* = 0), cartilage destruction (*n* = 1, 7.70%; *p* = 0.003), and crystal deposits (*n* = 0). **Conclusions:** The therapeutic approach for OA is a dynamic and intricate process, for which the type of affected joint and the underlying pathogenetic mechanism at a specific stage of the disease’s evolution is essential. MSK US is one of the options for the clinical phenotyping of OA. Some of the suggested ultrasound subtypes may serve as the rationale for selecting a particular treatment.

## 1. Introduction

Osteoarthritis (OA) is a chronic systemic disease that affects not only articulating joints but also the whole array of joint structures [1,2,3]. It is one of the most common chronic, socially significant diseases, linked with a decline in the quality of life of patients, social isolation in late-stage disease, and an enormous burden to healthcare systems around the world, caused by the constantly increasing cost of treatment [4,5]. Concomitant diseases lead to disability in a large portion of patients suffering from OA.

Studies in the field of OA are constantly being conducted in an attempt to characterize this disease in detail and increase the effectiveness of therapeutic strategies [6,7,8,9]. However, successful clinical trials meeting primary endpoints are rare, and OA remains one of the few rheumatic diseases without any established disease-modifying therapy [10,11]. One possible explanation is that researchers and clinicians still use ineffective tools for OA classification, patient stratification, and the assessment of disease progression [12]. OA is not a homogenous disease, and its clinical manifestation largely depends on its localization and the stage of the process. Although there is no established disease-modifying OA drug (DMOAD) therapy, clinicians are treating some aspects of the disease with a variety of pharmacological and non-pharmacological means. A personalized approach is key, and it is determined by the clinical phenotype of the disease, depending on the medical history, physical examination, and imaging techniques. Conventional X-ray remains the primary imaging modality in assessing the disease stage during the follow-up period, despite its lack of sensitivity for early cartilage changes (which already occur before radiographic joint space narrowing) and its ability to detect only late bone changes (osteophytes and joint space narrowing) [13]. In most cases, the changes that lead to the development of OA are located in soft tissue. These early modifications are detectable with magnetic resonance imaging (MRI) and musculoskeletal ultrasound examination (MSK US) [14,15,16,17,18,19]. OA is considered a disease of the whole joint—the joint itself as an organ and not only the articular cartilage. Symptoms can arise from the underlying bone, synovium, muscles, tendons, and ligaments or the entheses [20,21,22]. MRI provides not only detailed information about soft tissue and the underlying bone, thus having a crucial role in understanding the disease course and guiding future therapeutic options due to its ability to show the joint in its entirety, but also has a major role in clinical trials. Regardless of the sensitivity of the modality, MRI has its drawbacks—its cost and limited availability in daily practice deem it unsuitable for monitoring patients with OA [23]. For these reasons, it is not routinely used for evaluation and follow-up in patients with OA. Although the use of MSK US in clinical trials lags behind that of conventional radiography, there is increasing interest in this technique in daily practice. This imaging modality has numerous advantages, as US essentially has no contraindications for its implementation, can enable the dynamic visualization of the examined structures, and gives information about both inflammatory and structural changes that have occurred [24,25,26,27]. US can visualize hyaline cartilage, i.e., the surface of the subchondral bone, with changes such as erosions, osteophytes, and extra-articular soft tissue and intra-articular changes—namely synovitis and joint effusion [28,29,30,31]. Furthermore, in the hands of a trained clinician, it is an invaluable imaging marker in stratifying and characterizing the clinical phenotype of OA, thus influencing therapeutic decision-making.

The identification of different phenotypes of OA by US could be useful in identifying so-called rapid progressors. These are patients with a less favorable prognosis and more pronounced inflammation, who would be appropriate candidates for clinical trials [32]. US can select patients based on a clinical phenotype for inclusion in a clinical trial and predict the response to a particular therapy [10,33,34]. In summary, in everyday practice and for research purposes, US has diagnostic, prognostic, and therapeutic importance, especially when MRI is contraindicated, less appropriate, or unavailable due to various reasons.

This article aims to evaluate and summarize the potential of MSK US in the process of determining the clinical phenotype of OA and to emphasize the importance of this imaging modality in evaluating further therapeutic strategies.

## 2. Materials and Methods

This was a single-center prospective study conducted in the period of September 2023–June 2024 at the Rheumatology Clinic of University Hospital “Kaspela” and in outpatient care. Inclusion criteria: Consecutive patients with proven OA of the knee joint, OA of the hip joint, hand OA, and OA of the shoulder joint, according to fulfilled ACR diagnostic criteria, from grade 1 to 3 on the Kellgren–Lawrence scale, were included [35,36,37]. Written informed consent was taken from all participants in accordance with the Declaration of Helsinki, prior to laboratory, physical, and US examinations. Exclusion criteria were other musculoskeletal diseases—inflammatory joint diseases, connective tissue diseases, etc.—previous joint surgery procedures; a history of or current trauma; those with grade 4 OA on the Kellgren–Lawrence scale; and an unwillingness to participate in the study.

Each patient was examined by two experienced rheumatologists. Physical examination was followed by X-ray and MSK US, performed within two days.

### Ultrasound Assessment

US of the joints and periarticular structures, namely the knee, hip, and wrist joints and the small joints of the hands and shoulder joints, was performed by certified ultrasonographers (RK, ZB) with over 15 years of experience in MSK US, who were blinded to the radiographic findings, as well as to the therapeutic regimens. The ultrasonographic examination was performed according to the standardized and OMERACT-approved recommendations, and the established findings were classified and staged according to the OMERACT definitions of pathological changes. The ultrasound assessment of the wrists, knees, hip joints, and shoulders, including both the joints and periarticular structures, was conducted using a Logic E9 XDclear GE machine, Medical Systems Ultrasound and Primary Care Diagnostics LLC, 9900 Innovation Dr, Wauwatosa, WI, USA with an ML6-15-D matrix array linear probe and a hockey stick probe for the hands and fingers. A linear probe of 5–18 MHz was used for US scanning. The depth for the MCP joint was 2.5–3 cm, and the depth for the wrist was 3–4 cm. The settings for the power Doppler US (PDUS) were as follows: a pulse repetition frequency of 750 Hz, a low wall filter of 150 Hz, a scale of 3 cm/s, and a Doppler gain just below the color noise threshold for the visualization of the low-velocity flow. The articular cartilage of the metacarpal head was assessed in maximal flexion of the metacarpophalangeal joint to expose its surface to the US beam.

Based on the characteristic pathological changes in OA (Table 1), the articular and periarticular structures of the OA joint were examined using MSK US. Thus, this imaging modality allowed the identification of several main subtypes of OA, corresponding to the changes occurring in OA.

For the staging of the aforementioned changes and their follow-up over time, an ultrasound scoring system has been developed—the ultrasound OMERACT score [31,38]. According to these systems, the findings are scored as follows (Table 2).

## 3. Suggestions for US Phenotypes

Based on MSK US, we have differentiated several ultrasonographic phenotypes with their characteristic features:

- Predominant intra-articular effusion (with minimal synovial proliferation (synovial sacs) and large intra-articular effusion;
- Predominant synovial proliferation, fat pads, and Hoffa synovitis (with a small amount of intra-articular effusion);
- Predominant cartilage destruction (with almost no synovitis and intra-articular effusion);
- Altered subchondral bone (superficial bone erosions or osteophytes);
- Changes in extra-articular soft tissue (ligaments, tendons, capsule, entheses, muscles, bursae, Baker’s cysts, menisci—for knee joints); periarticular cystic lesions (including meniscal);
- Crystal deposits (MSU, CPPD);
- Post-traumatic (fractures, cruciate ligament, menisci—for knee joints, tendons).

## 4. Examples of US Phenotypes

**Figure 1:**
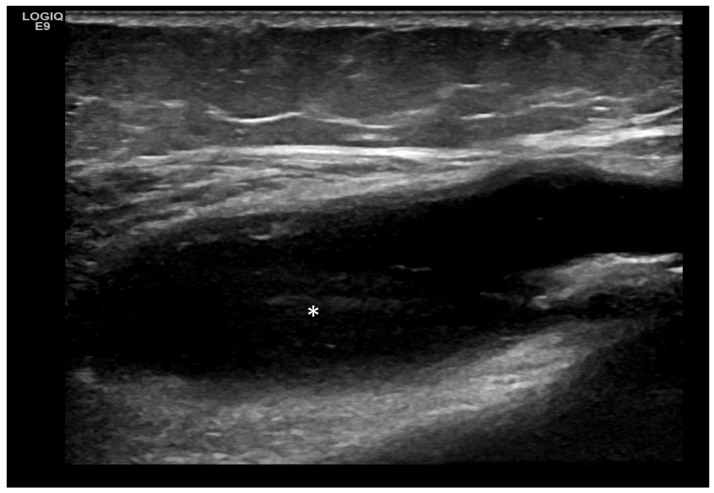
Significant amount of synovial effusion in the suprapatellar recess of the knee joint, presenting as an abnormal anechoic zone (asterisk). This US phenotypic expression corresponds to OA disease activity and progression.

**Figure 2:**
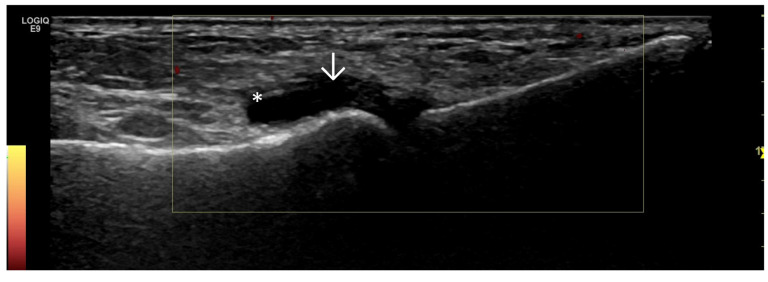
Longitudinal scan of the metatarsophalangeal joint. The intra-articular effusion is visible as an anechoic abnormal zone (arrow) and, more discretely, the synovial proliferation as a hypoechoic zone peripheral to it (asterisk).

Predominant Intra-Articular Effusion (With Minimal Synovial Proliferation (Synovial Sacs) and Large Intra-Articular Effusion (Figure 1 and Figure 2)

**Figure 3:**
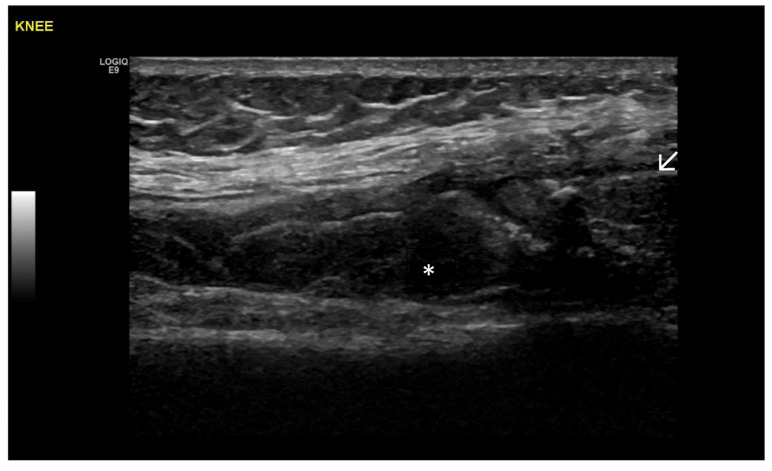
Ultrasonography of the knee joint—longitudinal scan at the level of the suprapatellar recess. Suprapatellar fad pad—arrow. There is pronounced synovial proliferation (asterisk) at the level of the suprapatellar recess, which presents as abnormal hypoechoic intra-articular tissue.

**Figure 4:**
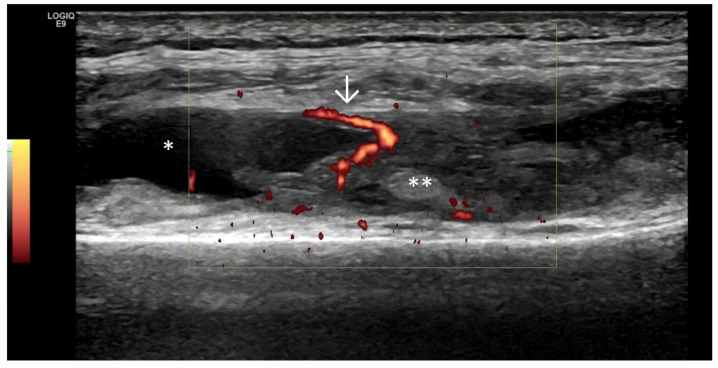
Ultrasonographic suprapatellar longitudinal scan of the knee joint. Against the background of the intra-articular hydrops (asterisk), which is presented as an anechoic zone, the hypoechoic synovial proliferation (two asterisks) with a positive Doppler signal (arrow), an indicator of inflammatory activity, is visible.

Predominant Synovial Proliferation With/Without Doppler Activity (With a Small Amount of Intra-Articular Effusion, Fat Pads, and Hoffa Synovitis) (Figure 3 and Figure 4)

**Figure 5:**
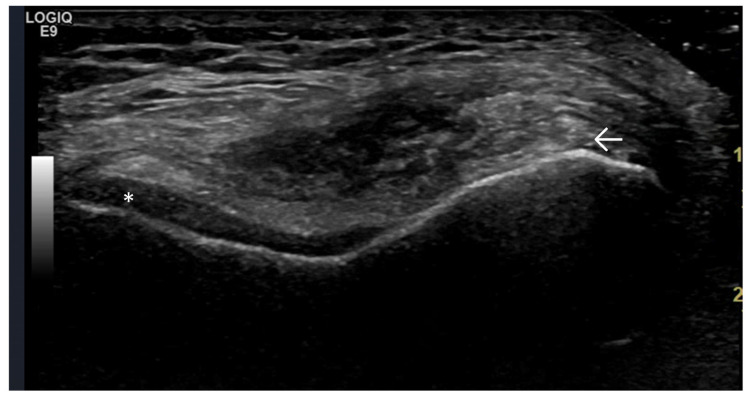
Transverse scan of the knee joint in full flexion for maximum visualization of the hyaline cartilage. An uneven reduction in the thickness of the hyaline cartilage is seen along the femoral condyles. The hyaline cartilage (*), which is laterally relatively preserved in terms of thickness, echogenicity, and homogeneity, presents as an anechoic zone above the hyperechoic bone contour. In the medial direction, it is completely reduced (arrow)—an expression of an advanced degenerative process.

Predominant Cartilage Destruction (Without or With Almost No Synovitis and Intra-Articular Effusion) (Figure 5)

**Figure 6:**
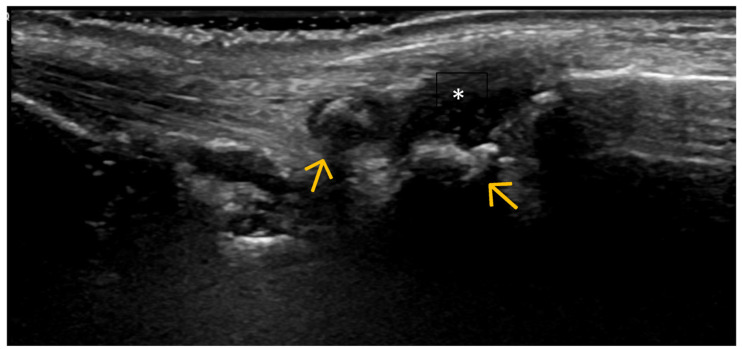
Volar US scan of the 1st carpometacarpal joint of a patient with OA at this level. Step-up bone defects are indicators of osteophytic formations (arrows); anechoic areas (asterisk) indicate a small amount of intra-articular fluid.

**Figure 7:**
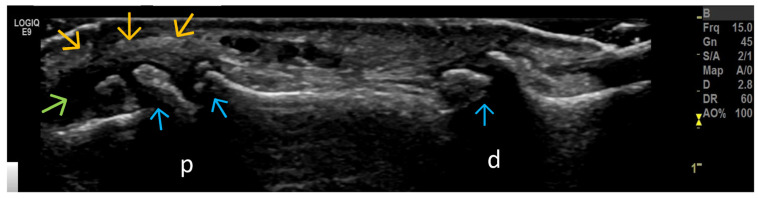
Longitudinal US scan of interphalangeal joints in a patient with hand OA. Hyperechoic step-up defects at the level of the proximal interphalangeal joint (PIP) (p) and distal interphalangeal joint (DIP) (d) correspond to osteophytes (blue arrows) that lead to the expansion of the joint capsule (outlined by orange arrows). Secondary synovitis is present (green arrow).

**Figure 8:**
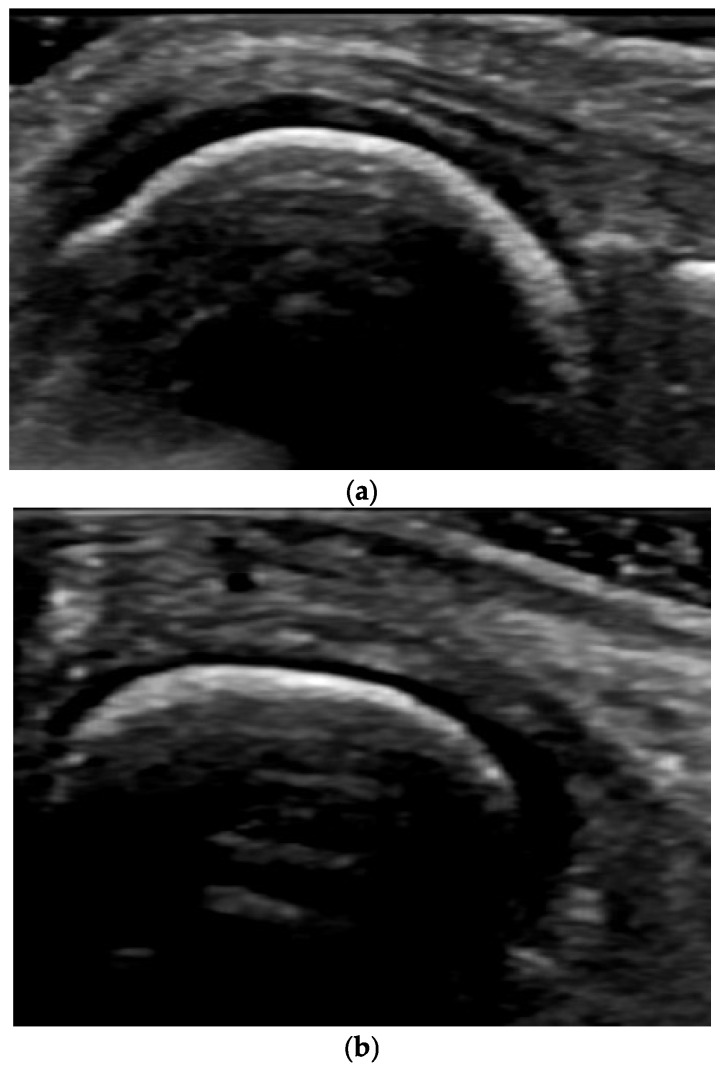
Longitudinal US scan of MCP joint in full flexion for assessment of hyaline cartilage. (**a**) Ultrasound image of a healthy subject. Hyaline cartilage is anechoic, homogeneous, and has a preserved thickness across the entire visible surface. (**b**) Various changes occurring in the joint with OA. Findings are established both on the cortical bone—thickening, irregularities, altered shape of the MC head—and on the hyaline cartilage—a change in homogeneity towards increased echogenicity with the progression of the OA process, a decrease in homogeneity, unclear demarcation of the surface edge of the cartilage, increased echogenicity of the inner edge, and a progressive reduction in cartilage thickness until its complete disappearance.

Altered Subchondral Bone (Superficial Bone Erosions or Osteophytes) (Figure 6, Figure 7 and Figure 8)

**Figure 9:**
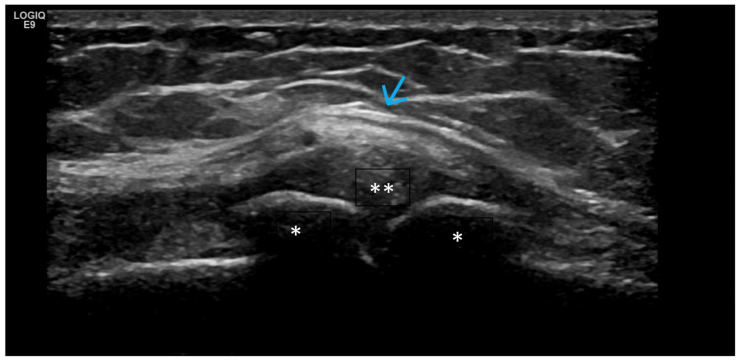
Longitudinal US medial scan of the knee joint. Extrusion of the medial meniscus (**) as a result of advanced structural changes from a reduction in hyaline cartilage and advanced osteophytosis (*) leads to its prominence towards the collateral ligament (blue arrow) and the thickening of the joint capsule as a result of the chronic OA process.

**Figure 10:**
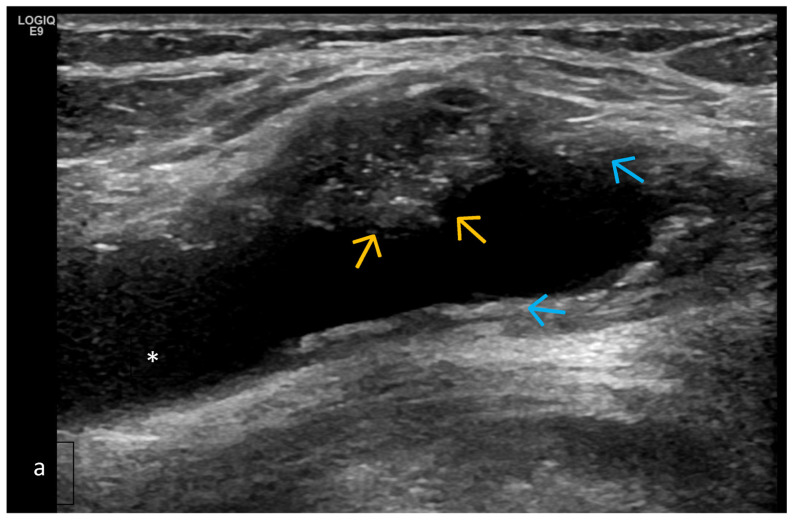

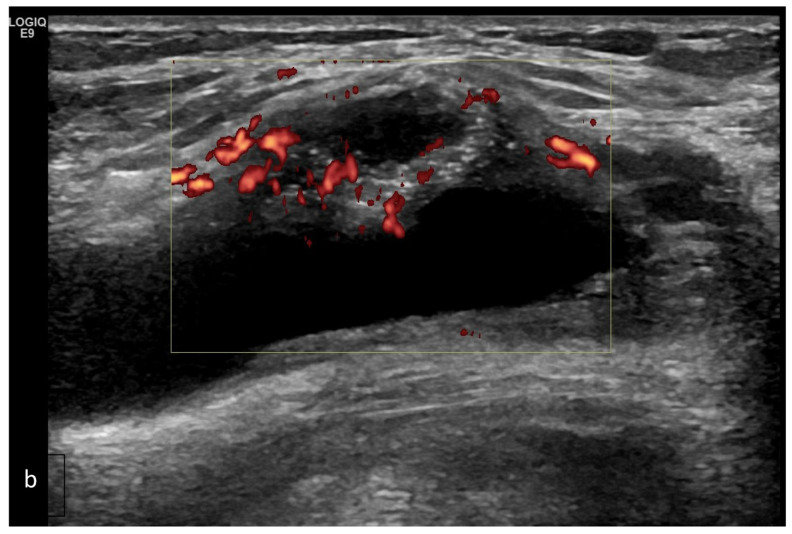
Posterior longitudinal US scan of the knee joint for the assessment of the popliteal fossa of a patient with knee OA. (**a**) Baker’s cyst with effusion (*), where the heterogeneous abnormal hypo-/hyperechoic mass is consistent with synovitis (blue arrows). Hyperechoic spots are an indicator of crystal deposition (orange arrows). (**b**) The same patient from (**a**). Power Doppler US modality reveals synovitis with a high degree of activity.

Changes in Extra-Articular Soft Tissue (Ligaments, Tendons, Capsules, Entheses, Muscles, Bursae, Baker’s Cysts, Menisci—For Knee Joints); Periarticular Cystic Lesions (Including Meniscal) (Figure 9 and Figure 10)

**Figure 11:**
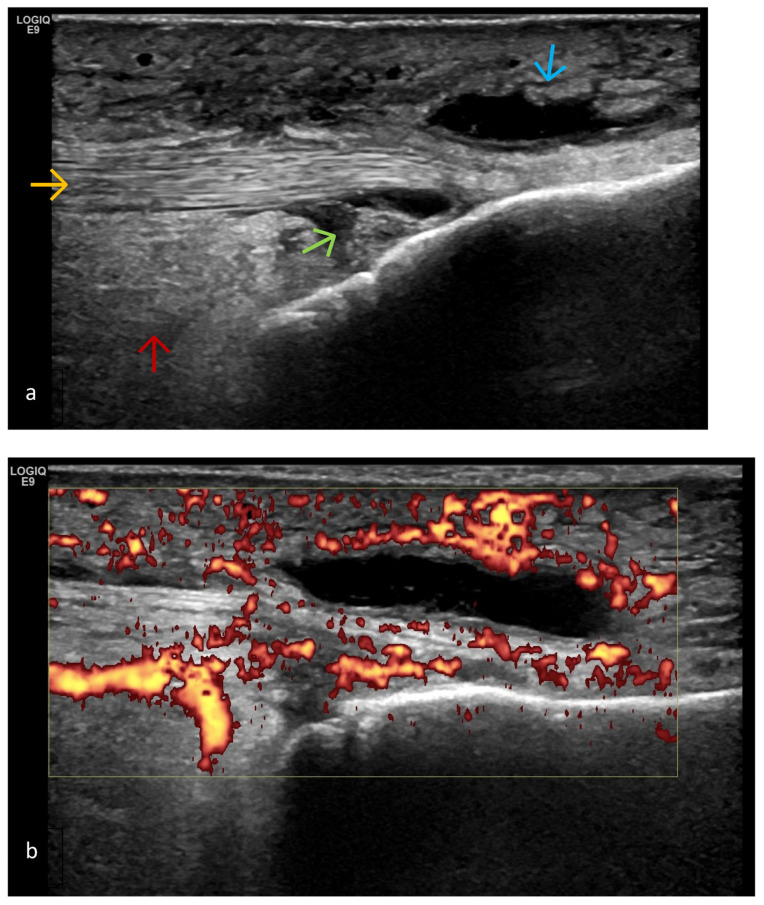
(**a**) Longitudinal infrapatellar US scan of a patient with knee OA—B-mode. The patellar tendon (orange arrow) is well traced distal to its insertion on the tibia. There is superficial (blue arrow) and deep (green arrow) infrapatellar bursitis with a mixed component—both proliferative with synovitis and exudative with exudate. Hoffa’s fat pad synovitis is also clearly visible (red arrow). (**b**) Power Doppler modality reveals highly active disease, grade 3.

Hoffa’s Fat Pad Synovitis (Figure 11)

Infrapatellar adipose tissue that is intracapsular and extrasynovial occupies the anterior compartment of the knee joint. Anteriorly, it is bordered by the patellar tendon and joint capsule, superiorly by the lower pole of the patella, inferiorly by the proximal tibia and deep infrapatellar bursa, and posteriorly by the synovial membrane. Because it is highly vascularized and innervated, it is a potential source of pain in this area of the knee joint.

**Figure 12:**
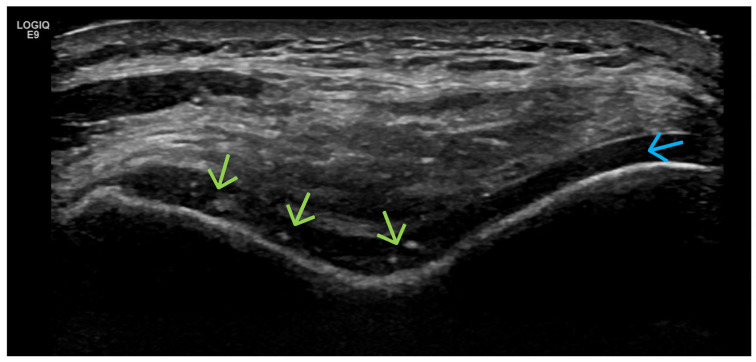
Transverse US scan of the knee joint in maximal flexion, allowing optimal visualization of the hyaline cartilage (V-shaped anechoic line (blue arrow) “covering” the hyperechoic bony contour of the femoral condyles). The presence of deposits of different sizes and increased echogenicity located in the middle of anechoic hyaline cartilage corresponds to deposits of calcium pyrophosphate CaPP (green arrows). The thickness of the hyaline cartilage appears relatively uniform throughout its sections.

**Figure 13:**
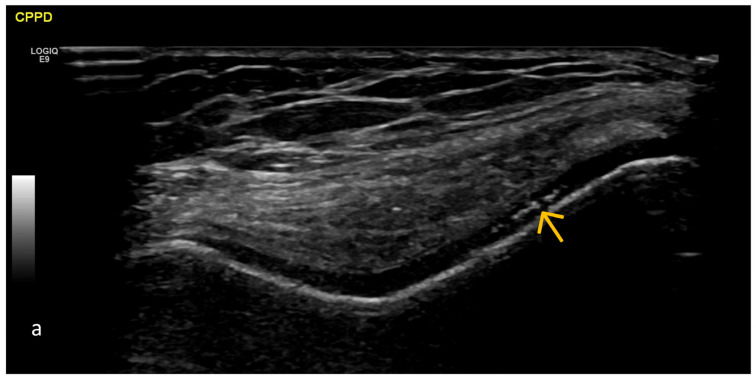

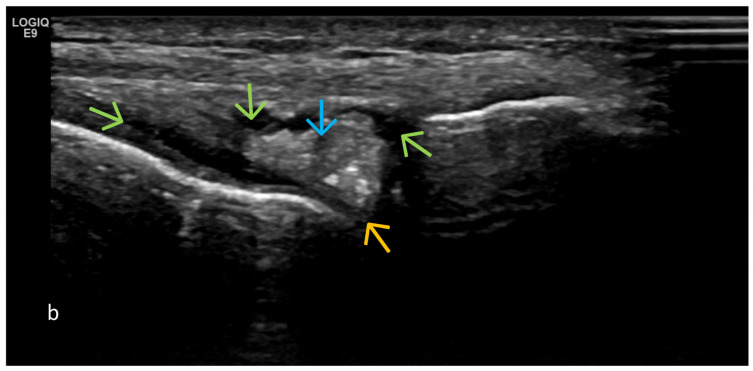
(**a**) US image from a transverse scan of the knee joint in maximal flexion illustrates the slight reduction in the thickness of the hyaline cartilage, as well as the change in its echogenicity. In addition, linear hyperechoic deposits of CaPP are found in the anechoic hyaline cartilage (orange arrow). (**b**) Ultrasonographic longitudinal scan of the knee joint medially in a patient with knee OA. The triangular fibrocartilage of the lateral meniscus presents with altered echogenicity and heterogeneity as a result of the pathological process, with a partial rupture of the meniscus—the hypo-anechoic line that almost completely separates the meniscus vertically (blue arrow). Hyperechoic spots in the fibrocartilage correspond to CaPP deposits (orange arrow). The anechoic zone surrounding it corresponds to hydrops in the lateral recess (green arrows).

**Figure 14:**
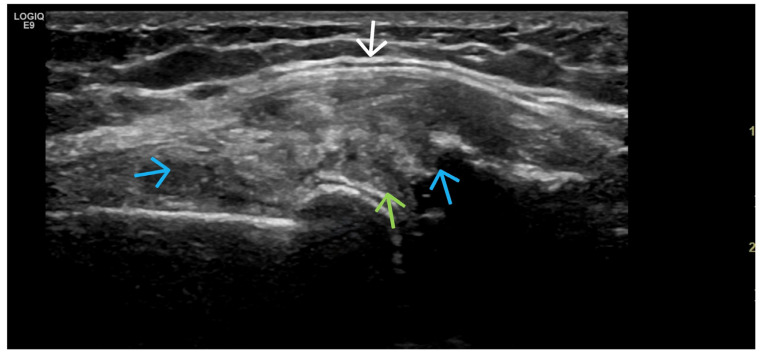
Longitudinal ultrasonographic scan of a patient with knee OA. The hyperechoic double contour is definitive evidence of the deposition of monosodium urate crystals on the surface of the hyaline cartilage. Double contour sign—green arrow. This finding is present in gout. Proximal to this finding, a heterogeneous mass of hyper-, hypo-, and anechoic tissue is found, as is characteristic of tophi, a sign of chronic gout (blue arrow). The mass, in parallel with the advanced OA process, leads to the dislocation of the collateral ligament (white arrow).

**Figure 15:**
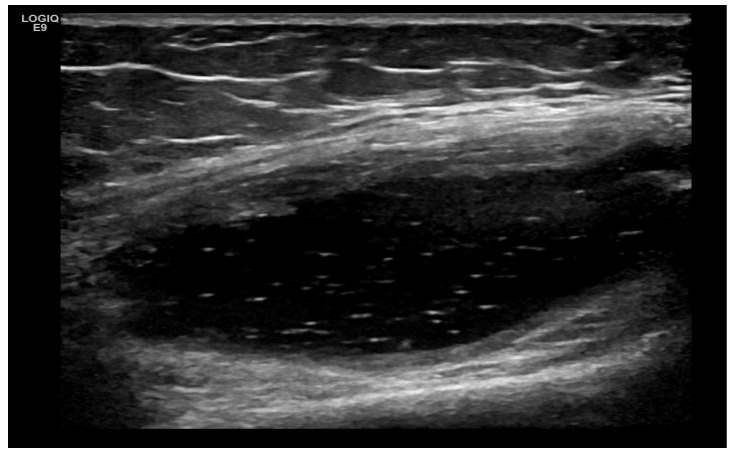
Ultrasound image of the so-called “starry sky” or “snowstorm” appearance in a suprapatellar recess in a patient with OA. Against the background of the anechoic US image of the hydrops, the hyperechoic particles in the middle of the liquid stand out well, which correspond to formed crystalline substances, established in crystalline arthropathy from monosodium urate.

**Figure 16:**
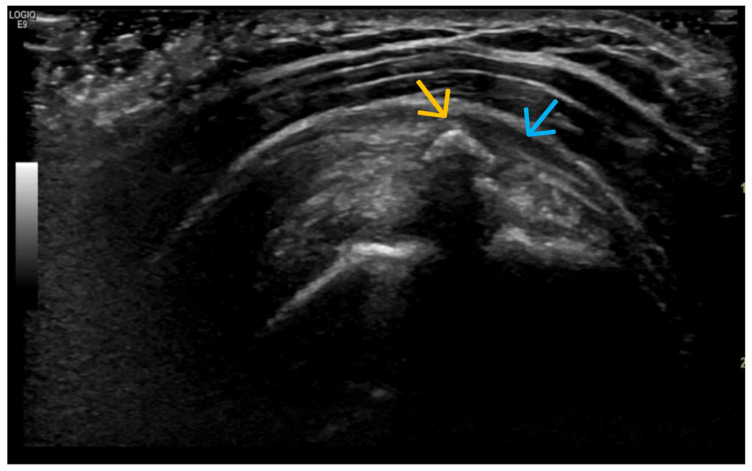
Ultrasonographic image of the shoulder (glenohumeral joint) with predominant visualization of the supraspinatus tendon in its longitudinal scan. The hyperechoic structure (orange arrow) in the middle of the tendon fibers, with the distally descending acoustic shadow, corresponds to a calcium-dense deposit. In addition to these findings, soft tissue changes are found—increased size and change in its echogenicity, i.e., tendinitis of the tendon, as well as discrete bursitis (blue arrow).

**Figure 17:**
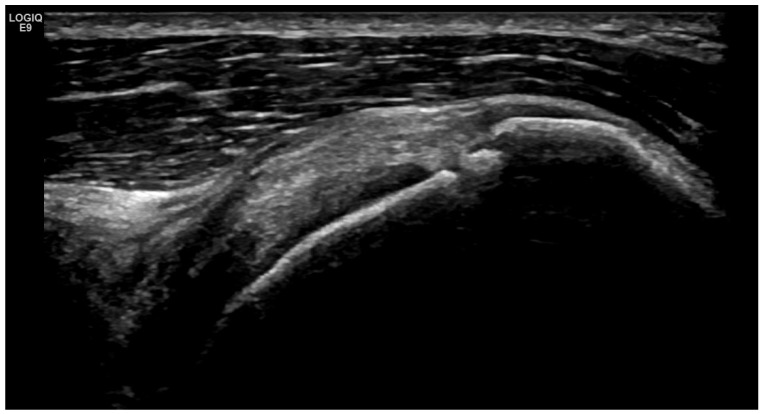
Ultrasound examination of a shoulder joint in a patient with omarthrosis. In parallel with the soft tissue changes at the level of the rotator cuff, a break in the bone contour is also found—a step-down defect corresponding to erosions.

Crystal Deposits (MSU, CPPD) (Figure 12, Figure 13, Figure 14, Figure 15, Figure 16 and Figure 17)

**Figure 18:**
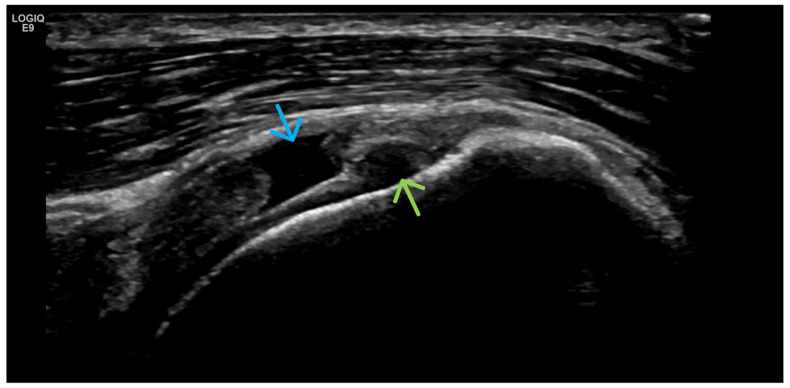
Anterior ultrasonographic scan of the shoulder joint of a patient with mechanical shoulder pain. Longitudinal US scan of the tendon of the supraspinatus muscle very clearly visualizes the partial ruptures of the tendon, which, through utilizing this imaging modality, are presented as anechoic zones—a partial rupture near the hyaline cartilage of the humerus (green arrow), as well as another in the middle of the bundles of tendon fibers (blue arrow).

**Figure 19:**
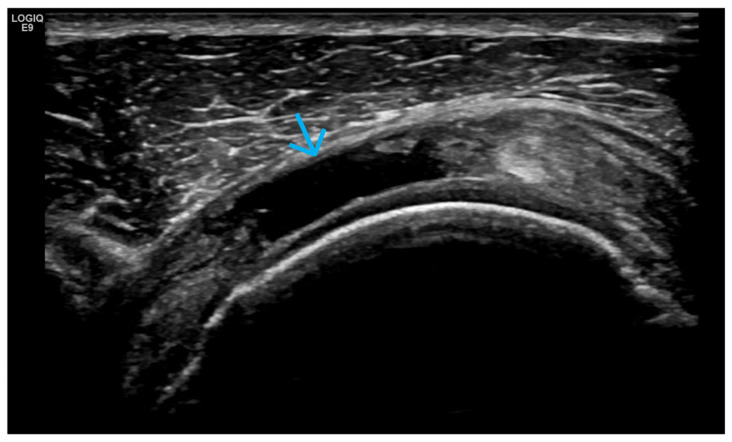
Anterior ultrasonographic scan of the shoulder joint in a patient with mechanical pain in the area due to a partial rupture of the rotator cuff. The large anechoic zone (blue arrow) corresponds to the loss of tendon fibers, resulting from their rupture.

**Figure 20:**
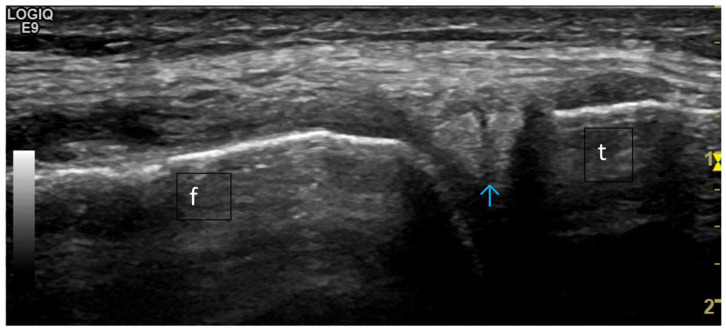
Ultrasonography of the knee joint—a longitudinal scan medially to visualize the medial meniscus. A complete rupture of the meniscus is presented, which passes through the triangular fibrocartilaginous tissue of the meniscus as a hypo-/anechoic line. f—femur, t—tibia; the blue arrow indicates the complete rupture of the medial meniscus.

Post-Traumatic (Fractures, Cruciate Ligaments, Menisci) (Figure 18, Figure 19 and Figure 20)

## 5. Statistical Analysis

The statistical program Minitab version 22.2.1 (2025) was used to analyze and graphically represent the data. The predominant and secondary phenotypes were tabulated for each OA localization and were presented as numbers and relative proportions (%). The rate of the most frequently occurring phenotypes was compared against that of the less frequent ones through paired z-tests to detect significant differences in their frequency. The initially acceptable type I error was set at 5%; however, it was further adjusted for the number of comparisons (Bonferroni).

## 6. Results

This study involved 259 patients who met the ACR diagnostic criteria for OA. Patients with OA of the knee joint constituted 123—80 women and 43 men. Meanwhile, 67 patients presented with OA of the hip joint—28 women and 39 men; OA of the hand joints was seen in 56 patients—41 women and 15 men; and 13 patients (6 women and 7 men) had OA of the shoulder joint. The basic clinical data for patients with OA are shown in Table 3.

### 6.1. Predominant US Phenotypes According to OA Localization

**Figure 21:**
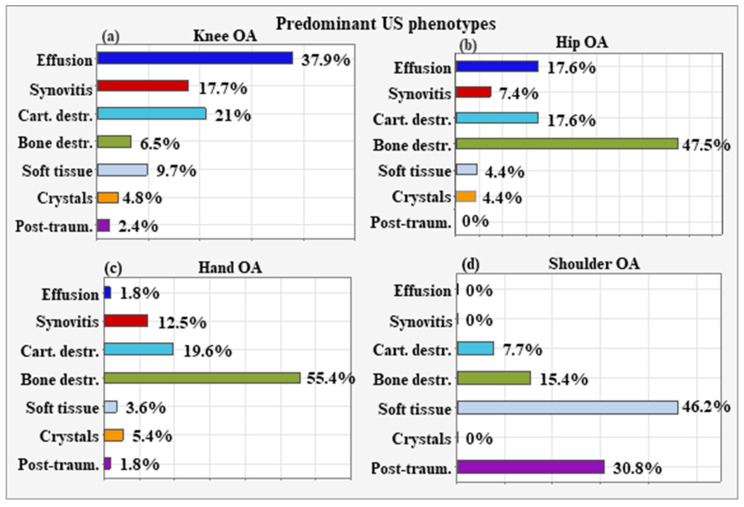
Predominant US phenotypes for OA localizations—Intraarticular effusion is the predominant US finding for Knee OA (**a**), altered subchondral bone is the main finding both for Hip OA (**b**) and Hand OA (**c**). For Shoulder OA, extra-articular soft tissue changes were the most frequent (**d**).

The most frequent predominant US phenotype for the patients with knee OA was intra-articular effusion (n = 47, 37.90%). The paired comparisons with z-tests showed that the proportion of patients with intra-articular effusion was significantly higher compared to the rest of the US phenotypes: synovial proliferation (n = 22, 17.70%; *p* < 0.001), cartilage destruction (n = 26, 21%; *p* = 0.001), altered subchondral bone (n = 8, 6.50%; *p* < 0.001), extra-articular soft tissue changes (n = 12, 9.70%; *p* < 0.001), crystal deposits (n = 6, 4.8%; *p* < 0.001), and post-traumatic (n = 3, 2.40%; *p* < 0.001) (Figure 21a).

The most common US phenotype for hip OA was altered subchondral bone (n = 32, 47.1%), which significantly differed from the other phenotypes: intra-articular effusion (n = 12, 17.60%; *p* = 0.001), synovial proliferation (n = 5, 7.40; *p* = 0.001), cartilage destruction (n = 12, 17.60%; *p* = 0.001), extra-articular soft tissue changes (n = 3, 4.40%; *p* = 0.001), crystal deposits (n = 3, 4.40%; *p* = 0.001), and post-traumatic (n = 0) (Figure 21b).

Altered subchondral bone was also the leading US phenotype for hand OA (n = 31, 55.40%), with significant differences compared to intra-articular effusion (n = 1, 1.80%; *p* < 0.001) synovial proliferation (n = 7, 12.50%; *p* < 0.001), cartilage destruction (n = 11, 19.60%; *p* < 0.001), extra-articular soft tissue changes (n = 2, 3.60%; *p* < 0.001), crystal deposits (n = 3, 5.40%; *p* < 0.001), and post-traumatic (n = 1, 1.80%, *p* < 0.001) (Figure 21c).

For shoulder OA, extra-articular soft tissue changes were the most frequent (n = 8, 46.20%), followed by post-traumatic (n = 4, 30.70%), as the rate of both phenotypes was significantly higher compared to that of the others: intra-articular effusion (n = 0), synovial proliferation (n = 0), cartilage destruction (n = 1, 7.70%; *p* = 0.003), and crystal deposits (n = 0) (Figure 21d).

### 6.2. Secondary US Phenotypes According to the Localization of OA

**Figure 22:**
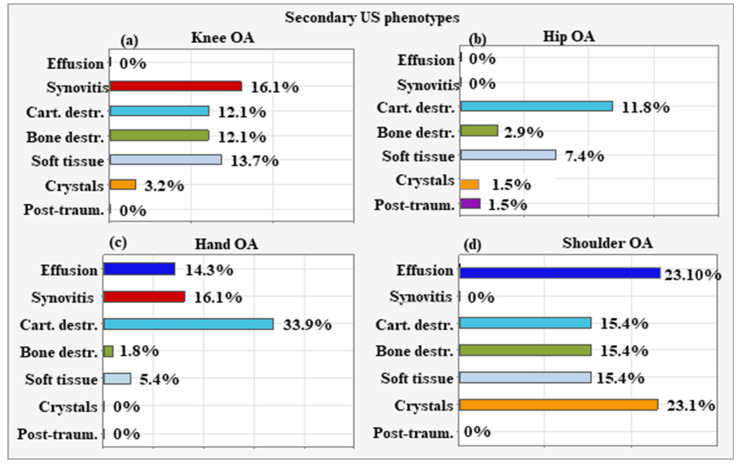
Secondary phenotypes for OA localizations—(**a**) Synovitis is the most common secondary US phenotype, followed by extra-articular soft tissue changes, cartilage destruction and altered subchondral bone for Knee OA. Cartilage destruction is the most frequent secondary US phenotype both for Hip OA (**b**) and Hand OA (**c**). For Shoulder OA, intra-articular effusion and crystal deposits have the highest presentation rate (**d**).

For knee OA, synovial proliferation was the most common secondary US phenotype (n = 20, 16.10%), followed by extra-articular soft tissue changes (n = 17, 13.70%), cartilage destruction (n = 15, 12.10%), and altered subchondral bone (n = 15, 12.10%). Overall, these three phenotypes were detected at similar rates, with no significant differences (*p* > 0.01 for all paired comparisons). Crystal deposits occurred at a significantly lower rate (n = 4, 3.20%) compared to the above three phenotypes (*p* ≤ 0.001 for all paired comparisons). Intra-articular effusion and post-traumatic changes were not detected (Figure 22a).

The most frequent secondary US phenotype for patients with hip OA was cartilage destruction (n = 8, 11.80%), followed by extra-articular soft tissue changes (n = 5, 7.40%), with no significant difference in the frequency of occurrence (*p* = 0.230). Changes in subchondral bone (n = 2, 2.90%), crystal deposits (n = 1, 1.50%), and post-traumatic changes (n = 1, 1.50%) were less frequent than cartilage destruction (*p* < 0.01 for all comparisons). Intra-articular effusion and synovial proliferation were not observed (Figure 22b).

The most common secondary US phenotype for hand OA was cartilage destruction (n = 19, 33.90%). It occurred more frequently than synovial proliferation (n = 9, 16.10%; *p* = 0.02), intra-articular effusion (n = 8, 14.30%; *p* = 0.015), extra-articular soft tissue changes (n = 3, 5.40%; *p* < 0.001), and changed subchondral bone (n = 1, 1.80%, *p* < 0.001). Crystal deposits and post-traumatic changes were not detected (Figure 22c).

For shoulder OA, intra-articular effusion and crystal deposits had the highest presentation rate, occurring in three patients each (23.10%). Next in frequency were cartilage destruction, altered subchondral bone, and extra-articular soft tissue changes, detected in two cases each (15.40%). Synovial proliferation and post-traumatic changes were not found. Due to the small sample size (n = 13), statistical comparisons were not performed for the secondary phenotypes associated with shoulder OA (Figure 22d).

## 7. Discussion

### Relationship Between OA Clinical Phenotype and Type of Underlying Affected Joint Region

There are numerous phenotypes described by different author collectives, but most of them consider only a certain aspect of intra-articular and peri-articular pathology in OA. For example, Dell’Isola et al. defined six clinical phenotypes of knee OA based on data available in the literature [39]: (1) chronic pain (with central mechanisms, e.g., central sensitization); (2) inflammatory (elevated levels of inflammatory biomarkers); (3) metabolic syndrome (high incidence of obesity, diabetes, and other metabolic disorders); (4) bone and cartilage metabolism (change in local tissue metabolism); (5) mechanical overload characterized primarily by varus deformity and medial compartment disease); (6) minimal joint disease characterized by minor clinical symptoms with slow progression over time. In addition to the six described clinical phenotypes, nine endotypes are also well known in OA of the knee joint [39,40]. A challenge for the future will be the identification of clinical phenotypes and the clear definition of their corresponding molecular endotypes [2]. Herrero-Beaumont et al. proposed four clinical phenotypes—biomechanical, osteoporotic, metabolic, and inflammatory—the characterization of which would help to accurately stratify OA patients in clinical trials [7]. The appropriate stratification of OA patients into certain phenotypes may optimize the design of individualized treatment regimens. To our knowledge, no study has been conducted to date to specify a therapeutic approach based on ultrasound phenotyping in patients with osteoarthritis. From a clinician’s perspective, in real-world clinical practice, the easiest way to differentiate between the clinical phenotypes in a given patient with OA is by imaging studies and primarily by MSK US. Based on this, we isolated several sonographic subtypes. This study clearly shows a combination of different US phenotypes for each OA localization, with one predominant phenotype (Figure 21) and one or several additional or secondary phenotypes (Figure 22). The most frequent predominant US phenotype for patients with knee OA was intra-articular effusion. Synovial proliferation was the most common secondary US phenotype, followed by extra-articular soft tissue changes, cartilage destruction, and altered subchondral bone. The predominant US phenotype for hip OA was altered subchondral bone. The most frequent secondary US phenotype was cartilage destruction, followed by extra-articular soft tissue changes. Altered subchondral bone was the leading US phenotype for hand OA as well. Cartilage destruction was the leading secondary US phenotype. It occurred more frequently than synovial proliferation, intra-articular effusion, extra-articular soft tissue changes, and changed subchondral bone. For shoulder OA, extra-articular soft tissue changes were prevalent. For shoulder OA, intra-articular effusion and crystal deposits had the highest presentation rates. The choice of a specific therapeutic approach should be determined by the US phenotype, with predominant changes at the level of the synovium, cartilage, bone, or periarticular soft tissue (Table 4).

According to Castaneda et al., OA is a heterogeneous progressive disease with different clinical phenotypes and affects all joint structures. OA is not a single entity but a heterogeneous syndrome with different clinical phenotypes that continuously evolve, eventually leading to common clinical manifestations. The clinical manifestation of a particular phenotype depends on the main underlying pathway and the predominant type of affected tissue (cartilage, menisci, synovial membrane, capsule, ligament, muscle, subchondral bone) [3]. In addition, we identify not only different clinical but also different ultrasonographic phenotypes depending on the leading pathologically altered joint structure.

Researchers in the field of OA focus mainly on studying knee OA [39,41,42], but OA of the hand and hip, which, in most cases, clinically manifests as a completely different disease entity, should not be ignored. The driving mechanisms of OA can vary greatly from joint to joint. For instance, OA of the hand is closest in its clinical manifestation to psoriatic arthritis (PsA) and rheumatoid arthritis (RA). The leading US finding for the predominant US phenotype of hand OA is bone destruction, followed by cartilage destruction and synovitis. The most common secondary US phenotype for hand OA is cartilage destruction, followed by synovial proliferation and intra-articular effusion. These findings support the notion that, although osteoarthritis was originally considered a degenerative disease, it is now generally accepted that low-grade inflammation is present in OA and plays a central role in its pathogenesis [42,43]. Moreover, persistent inflammation is a predictor of disease progression. For this reason, the modern understanding of OA considers it as a disease that affects the structures of the entire joint [1,6,44,45,46].

The rapid, easy, and reliable stratification of each patient, the correct recognition of the various OA phenotypes in the specific joint affected by the disease, and its objectification with image markers are of the utmost importance for clinicians in making the correct decision. Moreover, the entire process should take place at the patient’s bedside, based on a clinical examination, instrumental studies, and some easily available biological markers to facilitate this dynamic therapeutic process [24,47]. Imaging biomarkers have a leading role and great potential in diagnosis, staging, and the assessment of the rate of progression, as well as disease outcomes [16,48].

The clinical approach of the rheumatologist is flexible and is determined by the leading clinical phenotype at a given time point in the evolution of OA, which is dynamic and variable. Despite the controversial results regarding the success of new therapeutic options in OA, US phenotypes could rationalize therapy use, e.g., enabling the selection of patients who are more suitable for specific treatments.

## 8. Therapeutic Algorithm for OA

One of the great challenges of modern rheumatology is stratifying OA patients into different subgroups and validating an algorithm for phenotype-based therapy depending on this stratification. There is considerable heterogeneity across studies in the selection of participants and the methods used for research on phenotypes in osteoarthritis. The therapeutic strategy in OA is a complex and dynamic process, for which both the underlying pathogenetic mechanism (molecular endotype) and the type of the specific affected joint are important, as well as the imaging subtype at a given time in the specific affected joint at a given stage of disease evolution. A well-argued basis for choosing a specific therapeutic agent is the established US finding, i.e., the specific US subtype of OA, as the presence of synovitis and/or hydrops requires the use of intra-articular CS; active synovitis requires methotrexate; crystal deposits require colchicine; erosions require denosumab; dry joints require hyaluronic acid, glucosamine, etc. Based on the abovementioned MSK US phenotypes, a therapeutic algorithm for OA has been proposed.

## 9. Conclusions

OA is a heterogeneous progressive disease with varying clinical phenotypes, which depend on the most damaged tissue at a given point in time (bone, cartilage, synovium, joint capsule, ligaments, etc.). The natural evolution of the disease and its progression into the advanced stages leads to the homogenization of the symptoms. Therefore, it is of great importance to distinguish between clinical and pathogenetic phenotypes, especially in the early stages of the disease. Imaging modalities such as US help to determine the dominant tissue responsible for a particular symptom and the corresponding clinical picture in each patient with OA. Using US assessment in OA, the adequate stratification of these patients is possible by determining both the predominant and secondary US phenotype. Based on this information, the therapeutic decision (NSAIDs, SYSADOA, HA, PRP, corticosteroids, bisphosphonates, etc.) is ensured to be reasonable and precise. This is an example of subtype-based therapy in OA. Moreover, considering the fact that OA is a dynamic disease that changes in phenotypic expression, US is a useful imaging modality, both in the diagnostic process and in the follow-up period.

## 10. Future Directions

The existing disparity between research and real clinical practice is possibly rooted not only in the fact that OA is a heterogeneous disease in its nature, but also in failing to take into account the fact that it undergoes dynamic changes in its evolution and can change its subtype. This directly reflects the need for a flexible clinical approach. The most practical approach in daily clinical practice is the personal assessment of the attending clinician with clinical data and imaging biomarkers in combination with selected serum biomarkers. If a consensus is built to improve clinical phenotypes with some of the biochemical markers, several practical approaches (combination of clinical + imaging + biochemical) could be proposed. Based on these, clinicians can make personalized decisions, and researchers can use them in future clinical trials to develop new target molecules. To choose the correct combination (clinical + imaging + biochemical), some form of artificial intelligence may be needed. The use of novel radiomics features in diagnosing OA shows potential as a supportive tool to enhance clinicians’ decision-making [49]. Another modern digital service that increasingly relies on US examination is telemedicine, including teleconsultations, and various devices are offered to perform US, even by the patients themselves. The term “tele-ultrasonography” is now being introduced, which highlights the growing role of US as one of the most appropriate methods for remote imaging in OA [50,51].

## 11. Limitations

One limitation is that this study was single-center. In the future, it would be reasonable to conduct a multicenter study involving several centers in different countries and with other experts in the field of ultrasonography.

Another limitation concerns the physical characteristics of US and, in particular, the inability of the US beam to pass through bone structures. For this reason, changes in the depth of the subchondral bone cannot be analyzed, and only superficial bone defects, such as erosions and osteophytes, can be visualized and characterized.

## Figures and Tables

**Table 1:** Pathological changes occurring in osteoarthritis (OA) [30].

Targeted Structure	Grade, Type
1. Cartilage	0, I, II, III grade
2. Bone	Osteophytes (0, I, II, III grade) and erosions
3. Synovium	0, I, II, III grade
4. Synovial hypertrophy	+ or −
5. Doppler activity	With/without Doppler activity
6. Hoffa synovitis—degrees	For knee joint
7. Joint effusion	+ or −
8. Periarticular soft tissue	
9. Ligaments	
10. Bursae	
11. Menisci (extrusion, cysts)	For knee joint
12. Tendinopathy	
13. Entheses	
14. Compression of peripheral nerves	
15. Deposition in the joint and periarticular soft tissue	Monosodium urate crystals (MSU), calcium pyrophosphate (CPP) crystals

**Table 2:** Ultrasound OMERACT Scores [31,38].

Synovitis grade (0–3) Synovial hypertrophy (+/−) Effusion (+/−) PD (+) Cartilage grade (0–3) Osteophyte grade (0–3) Meniscal extrusion grade (0–2)

**Table 3:** Basic clinical data for patients with osteoarthritis.

	KOA (*n* = 123)	Hip OA (*n* = 67)	Hand OA (*n* = 56)	Shoulder OA (*n* = 13)
Gender (M/F)	43/80	39/28	15/41	7/6
Age (±SD), years	58.1 ± 6.7	61.3 ± 5.2	48.7 ± 3.9	49.8 ± 4.5
Duration of disease, years	7.6 ± 5.5	5.5 ± 6.7	6.3 ± 4.6	5.0 ± 4.1
BMI median, kg/m^2^	28.1 (24.5–34.6)	27.5 (23.2–34.7)	24.2 (19.8–26.6)	23.5 (20.1–27.3)
ESR (mm/h)	13.8 (5–42)	14.2 (6–28)	16.9 (10–44)	12.7 (8–22)
CRP (mg/L)	3.4 (1.1–7.2)	2.8 (1.0–5.2)	4.4 (2.8–6.2)	2.2 (0.8–5.3)

**Table 4:** MSK US subtypes and suggestions for therapeutic intervention based on them.

MSK US Subtype	Therapeutic Approach
**Predominant synovial proliferation (with a small amount of intra-articular effusion)**	NSAIDs (local and systemic) Physical therapy Intra-articular corticosteroids and/or methotrexate if unresponsive
2. **Predominant intra-articular effusion (with minimal synovial proliferation)**	Arthrocentesis with evacuation of synovial fluid and subsequent administration of intra-articular corticosteroids
3. **Predominant cartilage destruction (with almost no synovitis and intra-articular effusion)**	SYSADOAs (including glucosamine, chondroitin, diacerein, avocado soybean unsaponifiables (ASU)) Intra-articular hyaluronic acid Intra-articular platelet-rich plasma (PRP)
4. **Altered subchondral bone (superficial bone erosions or osteophytes)**	Bisphosphonates Denosumab Methotrexate—for hand OA
5. **Changes in extra-articular soft tissue (ligaments, tendons, capsules, entheses, muscles, bursae, Baker’s cysts)**	Aspiration of Baker’s cysts and application of CS CS infiltration around extra-articular soft tissue PRP infiltration around ligaments and tendons
6. **Crystal deposits (MSU, CPPD)**	NSAIDs Colchicine
7. **Post-traumatic (fractures, cruciate ligaments, menisci)**	NSAIDs and SYSADOAs (glucosamine, chondroitin, diacerein, avocado soybean unsaponifiables (ASU)) Intra-articular hyaluronic acid Intra-articular platelet-rich plasma (PRP)

## Data Availability

All the data are securely stored in a database that complies with the institutional data protection standards at University Hospital “Kaspela”.
